# Influence of gut microbiota and immune markers in different stages of colorectal adenomas

**DOI:** 10.3389/fmicb.2025.1556056

**Published:** 2025-04-16

**Authors:** Xianmei Wang, Hang Chen, Meng Yang, Minshan Huang, Dan Zhang, Mingke Li, Hui Wang, Qingqing Zhou, Lihong Lu, Yu Li, Jiangkun Yu, Lanqing Ma

**Affiliations:** ^1^Yunnan Institute of Digestive Disease, The First Affiliated Hospital of Kunming Medical University, Kunming, China; ^2^State Key Laboratory for Conservation and Utilization of Bio-Resources in Yunnan, School of Life Sciences, Yunnan University, Kunming, Yunnan, China

**Keywords:** colorectal adenoma, colorectal cancer, microbiota, PD-L1, IL-6, IFN-γ

## Abstract

**Objective:**

Colorectal adenomas (CRA) are the primary precancerous lesions leading to colorectal cancer (CRC). Early detection and intervention of CRA can significantly reduce the incidence of CRC. We investigated the relationships between the gut microbiome and the expression levels of PD-L1, IL-6, and IFN-γ at different CRA stages.

**Methods:**

Participants were divided into normal, non-advanced adenoma (NAA), and advanced adenoma (AA) groups. PD-L1 expression in collected tissues was analyzed via immunohistochemistry (IHC) and Western blotting. Serum IL-6 and IFN-γ levels were measured using Enzyme-Linked Immunosorbent Assay (ELISA). 16S rRNA gene sequencing was used to examine gut microbiota changes, with correlation analysis to assess microbial influences on CRA progression.

**Results:**

The main differences in bacterial composition among the three groups were found within the Firmicutes and Bacteroidetes phyla. In the normal vs. NAA comparison, *Clostridium sensu stricto, Faecalimonas, Gemmiger, and Ruminococcus* were more abundant in the normal group, while *Solobacterium* was enriched in the NAA group. For the normal vs. AA comparison, the normal group was enriched with *Anaerostipes, Blautia, Clostridium sensu stricto, Intestinibacter, Phocaeicola, and Turicibacter*, whereas *Solobacterium* was more abundant in the AA group. In the NAA vs. AA comparison, the NAA group exhibited higher levels of *Blautia, Faecalimonas, and Turicibacter* relative to the AA group. *Anaerostipes and Blautia* are positively correlated with *taurine and hypotaurine metabolism, propanoate metabolism, and zeatin biosynthesis.* PD-L1 protein levels progressively increase with CRA advancement. Additionally, *Faecalimonas, and Solobacterium* were negatively associated with IFN-γ, while *Gemmiger, and Anaerostipes* were positively associated with IL-6.

**Conclusion:**

This study highlights the dynamic alterations in gut microbiota composition and their potential influence on the regulation of inflammatory cytokines and PD-L1 expression during CRA progression. The enrichment of protective taxa, such as *Anaerostipes* and *Blautia*, in the normal group emphasizes their potential role in mitigating adenoma progression. Dietary modulation to promote the proliferation of these beneficial bacteria could serve as a promising strategy to improve colorectal health. Future research should further explore the specific relationships between dietary components, gut microbiota, and metabolic pathways, and assess the effects of dietary interventions on gut health.

## 1 Introduction

Colorectal cancer (CRC) is the third most common malignant tumor globally and the second leading cause of cancer-related death, accounting for approximately 10% of all cancer-related deaths (Sung et al., 2021). In China, CRC has become the third most common malignant tumor (Zheng et al., 2019). Over 85% CRCs originate from colorectal adenomas (CRA), and the causal relationship between the two has been established (Fu et al., 2025). The “normal mucosa-adenoma-cancer” pathway is considered the main route for CRC formation (Mang, 2019). The reported prevalence of CRA in China is approximately 13.3%–18.1%, with the prevalence of advanced adenomas being about 3.5% (Hong et al., 2018; Pan et al., 2020). Advanced adenomas are significant precursors to CRC, with the risk of progression to CRC increasing significantly by approximately 30%–50% (Conteduca et al., 2013). Although much research has focused on the metastatic and therapeutic aspects of advanced CRC, studies on the CRA stage are relatively limited, particularly in the areas of gut microbiota and dietary influences on CRA. Therefore, this study aims to explore the biomarkers and microbiota changes during the CRA stage, combined with dietary interventions, to provide new insights for early diagnosis and intervention of CRA.

Dietary interventions, as a non-pharmacological treatment approach, have been widely applied in the management of various diseases. In the prevention and treatment of CRA and CRC, a well-balanced diet can significantly reduce disease risk and improve patient prognosis. Studies have shown that the intake of dietary fiber can lower the risk of CRC by increasing the abundance of beneficial gut microbiota and reducing carcinogens in the gut (Munteanu and Schwartz, 2024; Song et al., 2018). Therefore, dietary treatment holds great promise as an effective measure for preventing the progression of CRA and the occurrence of CRC.

The “normal mucosa-adenoma-cancer” process is a multifactorial and multistep complex process. The human gut microbiota, primarily composed of bacteria from the Firmicutes, Bacteroidetes, Actinobacteria, and Proteobacteria phyla, is essential for functions such as nutrient absorption and immune regulation (Adak and Khan, 2019). Dysbiosis, or imbalance in this microbiota, can disrupt these functions and contribute to the development of diseases like colorectal tumors. Specifically, dysbiosis is linked to CRA and CRC, potentially by damaging intestinal epithelial cells, altering metabolism, and producing carcinogens (Flemer et al., 2017; Sepich-Poore et al., 2021). This disruption may lead to abnormal crypt foci, promoting the formation of colorectal polyps and eventually cancer (Liang et al., 2020; Zouggar et al., 2020).

In the tumor microenvironment, pro-inflammatory cytokines like IFN-γ and IL-6 can upregulate PD-L1, a protein that helps tumors evade immune detection (Lim et al., 2016). This upregulation allows cancer cells to avoid being targeted by the immune system. The PD-1/PD-L1 pathway, increasingly recognized for its role in CRC, suppresses cytotoxic lymphocyte activity, facilitating immune escape (Chen et al., 2017; Wu et al., 2022). Additionally, changes in the gut microbiota composition and metabolism can contribute to CRC by influencing local inflammation and genotoxic stress (Cao et al., 2022). However, the exact relationship between PD-L1 expression (Freitas et al., 2021; Gatenbee et al., 2022; Miller et al., 2018) and plasma inflammatory cytokines (Huang et al., 2019; Shi et al., 2015) with disease progression remains unclear.

Our study aims to investigate changes in the gut microbiota, IL-6, IFN-γ, and PD-L1 across different stages of CRA. By identifying significant biomarkers associated with these stages, and regulating gut microbiota composition and the production of beneficial metabolic products through dietary interventions.

## 2 Materials and methods

### 2.1 Ethics statement

This study was approved by the Ethics Committees of the First Affiliated Hospital of Kunming Medical University (project license number: L-26/2022). It was conducted in accordance with the principles of the Declaration of Helsinki. Written informed consent was obtained from all participants included in the study. Additionally, all research activities were carried out following applicable guidelines and regulations.

### 2.2 Subjects

**FIGURE 1:**
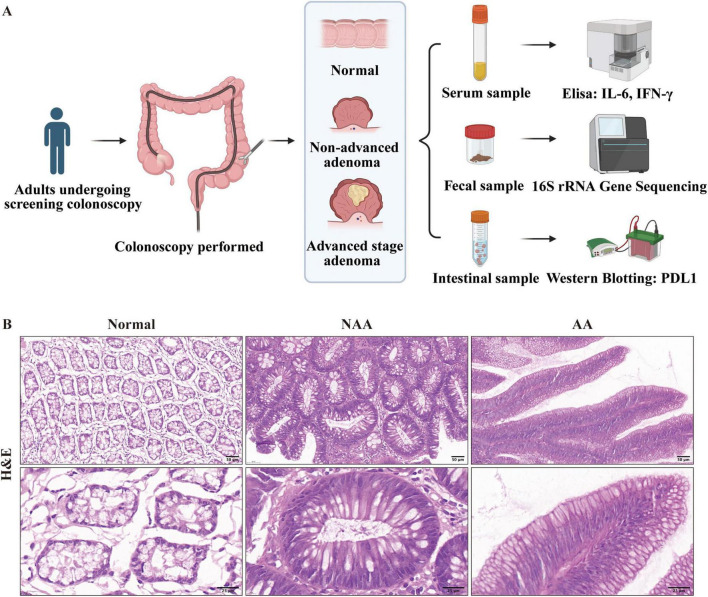
Cohort enrollment flowchart and Representative Hematoxylin and Eosin. **(A)** Cohort enrollment flowchart (provided by BioRender.com). **(B)** Representative Hematoxylin and Eosin (H&E) stained sections from each of the normal, NAA, and AA groups (30×, 100× magnification; scale bar: 50, 25 μm). Normal, normal group; NAA, non-advanced adenoma group; AA, advanced stage adenoma group.

The study was conducted between 2022 and 2023 at the First Affiliated Hospital of Kunming Medical University, with a total of 30 participants involved. These individuals were categorized into three groups based on their colorectal health status: normal (*n* = 10), non-advanced adenoma (NAA, *n* = 10), and advanced stage adenoma (AA, *n* = 10). Each participant provided three types of samples: serum samples to measure inflammatory cytokines IL-6 and IFN-γ via Enzyme-Linked Immunosorbent Assay (ELISA), fecal samples for analyzing gut microbiota composition using 16S rRNA gene sequencing, and intestinal tissue samples for assessing PD-L1 protein expression through Western blotting (Figure 1A). According to the 2020 guidelines by the US Multi-Society Task Force on the endoscopic identification and management of malignant colorectal polyps[6], CRA were classified based on their pathological structural characteristics determined by Hematoxylin and Eosin (H&E) staining. These categories included tubular adenomas (villous component < 25%), villous adenomas (villous component > 75%), and tubulovillous adenomas (villous component between 25 and 75%). For classification purposes, normal samples displayed regular colorectal mucosa, NAA samples exhibited tubular adenomas with a gross specimen length of <10 mm, a villous component <25%, and no high-grade dysplasia, while AA samples showed tubulovillous adenomas with a villous component ranging from 25 to 75% (Figure 1B). Participants were included in the study if they were diagnosed with one of the three groups based on H&E staining (normal, non-advanced adenoma, or advanced stage adenoma). The exclusion criteria consisted of individuals with a history of colorectal polyps or colorectal surgery, a personal or family history of gastrointestinal tumors, inflammatory bowel disease, infectious diseases, metabolic disorders (e.g., diabetes, obesity, and hyperlipidemia), immune system diseases, or severe hepatic or renal conditions.

### 2.3 Sample collection

All participants provided two samples of normal colonic mucosa or post-operative specimens, with the exact sampling locations detailed in Supplementary Table 1. One sample was stained with H&E, while the other was preserved in liquid nitrogen for subsequent PD-L1 protein expression detection through Western Blot and Immunohistochemistry (IHC). Prior to taking intestinal cleansing agents, fecal samples (5–10 g) were collected using single-use fecal sampling tubes, quickly frozen in liquid nitrogen for 16S rRNA sequencing. Blood samples (5 ml) were drawn from peripheral veins before colonoscopy, processed by centrifugation (3,000 rpm for 10 min at room temperature), and the serum was promptly frozen in liquid nitrogen for subsequent analysis of IL-6 and IFN-γ levels using ELISA.

### 2.4 Histology

Evaluated the expression of PD-L1 using IHC. Colorectal tissues were processed by embedding in OCT compound and then rapidly frozen in liquid nitrogen. These tissues were then sectioned into 5 μm slices. Tissue sections were baked overnight in an oven, dewaxed progressively with xylene and anhydrous ethanol, subjected to antigen retrieval with sodium citrate, cooled at room temperature, processed for membrane breakage, and blocked with sheep serum for 30 min. The sections were then incubated overnight with primary antibody, rewarmed for 1 h, incubated with secondary antibody, treated with horseradish peroxidase, visualized by DAB, dehydrated, and sealed. Colorectal sections underwent hematoxylin and eosin (H&E) staining for further examination. Image analysis was performed using ImageJ software.

### 2.5 Examination of inflammatory factors, and PDL1 level

The levels of serum inflammatory factors (IL-6, and IFN-γ) were determined using a commercial ELISA kit (IL-6, EK106/2-96; IFN-γ, EK180-96, Lianke Biotechnology Co., Ltd., Hangzhou, China) following the manufacturer’s instructions. Evaluated the protein expression of PD-L1 in colorectal tissue using Western blot. Fresh Colorectal tissues were pulverized under liquid nitrogen, and high-efficiency RIPA lysate (catalog number 89900, Thermo) was utilized for protein extraction. The resultant protein samples were then loaded (20 μg/10 μl per well) and resolved via sodium dodecyl sulfate-polyacrylamide gel electrophoresis (SDS-PAGE) on gels comprised of either 10 or 12.5% acrylamide. Subsequently, the separated proteins were transferred onto polyvinylidene fluoride (PVDF) membranes (catalog number ISEQ00010, Millipore). The membranes were then blocked with 5% skim milk in TBST for 2 h at room temperature. Following blocking, the membranes were incubated with primary antibodies targeted against PDL1 (1:1000) and GAPDH (1:1000). After thorough washing and incubation with appropriate secondary antibodies, the blots were visualized using a ChemiDoc XRS imaging system. The quantification of target proteins was achieved by normalizing their relative intensities in the experimental groups to those in the control group.

### 2.6 DNA extraction and 16S rRNA gene sequencing

Genomic DNA was extracted from fecal samples and the quality and concentration of purified DNA was measured with NanoDrop 2000. The isolated DNA was stored at −20°C for 16S amplified library preparation or qPCR. The hypervariable V3-V4 region of the 16S rRNA gene in 20 ng of DNA was amplified using gene-specific primers:5′ACTCCTACGGGAGGCAGCAG3′, R:5′ GGACTACHVGGGTWTCTAAT 3′. PCR conditions were 95°C for 3 min, 27 cycles of 95°C for 30 s, 55°C for 30 s and 72°C for 45 s, 72°C for 10 min and holding at 4°C. Two steps of amplification were performed to prepare the sequencing library using TransGen AP221-02. Quantification was performed using the QuantiFluor-ST™ (Promega), followed by paired-end sequencing on an Illumina platform.

### 2.7 16S rRNA gene amplicon sequencing analysis

Fastq files from the Illumina MiSeq run were processed through the QIIME2 pipeline as shown by Bolyen et al. (2019) using the DADA2 plugin (Callahan et al., 2016) to generate amplicon sequence variants (ASVs). Taxonomic classification was performed using the rdp_16S_v18_classifier database (Hung et al., 2022). While genus-level classifications are robust due to the resolution of the V3-V4 region of the 16S rRNA gene, species-level annotations were inferred based on probabilistic matches in the reference database. We performed multivariate testing between treatment groups and further performed *post hoc* comparisons when significant differences were observed. We determined alpha diversity indices: Pielou’s evenness, Shannon diversity and Observed species. Beta diversity was calculated based on the unweighted unifrac distance metric and visualized using principal co-ordinates analysis (PCoA).

### 2.8 Linear discriminant analysis and function predict

We identified taxa differentiating the normal, NAA, and AA groups using linear discriminant analysis effect size (LEfSe). LEfSe utilizes a non-parametric Kruskal-Wallis rank sum test to assess differential features with significantly different abundances between assigned taxa and performs LDA to estimate the effect size of each sequence variant as reported by Segata et al. (2011). LDA scores ranking differential taxa are displayed on a LEfSe bar chart according to their effect size. For LEfSe analysis data are first converted to log10 before the non-parametric Kruskal-Wallis rank sum test. The cut-off of the LDA score was set to 2, and significant features were considered with *P*-values lower than 0.05. To predict the metabolic pathways, phylogenetic investigation of communities by reconstruction of unobserved states (Picrust2) was performed from sequencing data, as shown before (Douglas et al., 2020). Fastq sequence files for each sample were processed using QIIME2. Representative sequences were used as input files for the Picrust2 analysis pipeline. Metabolic pathways were assigned based on the Kyoto Encyclopedia of Genes and Genomes (KEGG) (Langille et al., 2013).

### 2.9 Statistical analyses

Statistical evaluations in this study were conducted utilizing GraphPad Prism (ver. 9) and R (ver. 4.3.0). The microbial composition at different levels, KEGG as well as indices of alpha diversity were analyzed using Kruskal-Wallis test. Beta diversity was calculated using unweighted UniFrac distance, and a permutational multivariate analysis of variance (PERMANOVA) (Anderson, 2001) was performed in QIIME2 to confirm significant differences between groups. Spearman’s rank correlation method was used to analyze relationships between variables. To ensure consistency, data were preprocessed through normalization and scaling of variables before analysis. The data are presented as the mean ± standard error of the mean (SEM). Differences were deemed statistically significant at a threshold of *p* < 0.05.

## 3 Results

### 3.1 Microbial diversity analysis

#### 3.1.1 Alpha diversity

**FIGURE 2:**
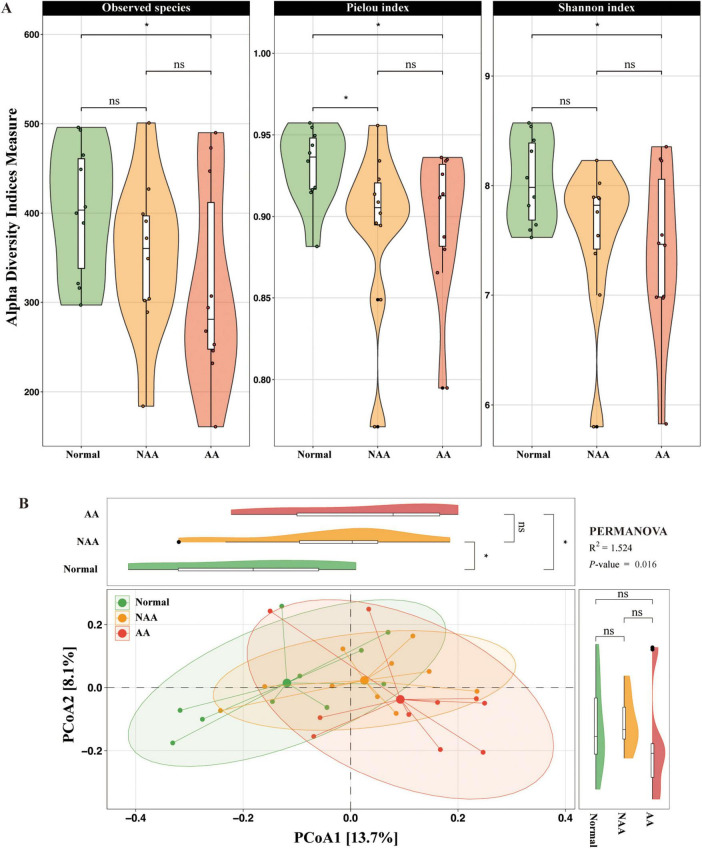
Gut microbiota profiling in normal, NAA, and AA groups. **(A)** Alpha diversity of microbial communities in normal, NAA, and AA groups, including observed species, Pielou index and Shannon index. **(B)** Coordination plot of principal co-ordinates analysis (PCoA) of microbial communities among normal, NAA, and AA groups. Normal, normal group; NAA, non-advanced adenoma group; AA, advanced stage adenoma group. The data are presented as means ± SD, *n* = 10. *P < 0.05.

The study examined changes in α-diversity and β-diversity of the gut microbiota across different stages of CRA using 16S rRNA gene sequencing. For alpha diversity, the observed species showed a slight decrease from the normal group to the NAA group and the AA group, with no statistically significant differences (ns) between the groups, except for a significant reduction (*p* < 0.05) between the normal and AA groups. The Pielou index was significantly higher in the normal group compared to the NAA and AA groups (*p* < 0.05). The NAA group exhibited the lowest Pielou index, while the AA group showed a slightly higher value compared to the NAA group, with no statistically significant difference (ns). The Shannon index progressively decreases from the normal group to the NAA group and further to the AA group, with a significant difference (*p* < 0.05) observed between the normal and AA groups. No statistically significant differences (ns) were observed between the normal and NAA groups or between the NAA and AA groups (Figure 2A).

#### 3.1.2 Beta diversity

Principal co-ordinates analysis was performed at the species level to investigate the gut microbial community structure, revealing significant differences among the normal, NAA, and AA groups (PERMANOVA, *R*^2^ = 1.524, *P*-value = 0.016) (Figure 2B). PCoA1 explained 13.7% of the variation in the microbial community structure, while PCoA2 accounted for 8.1%. These results highlight distinct microbial community profiles across the three groups, with a significant shift from normal to NAA through AA.

### 3.2 Differential analysis of gut microbiota composition

The LEfSe algorithm was employed to pinpoint the key bacterial taxa responsible for the differences in gut microbiota across the normal, NAA, and AA group phenotypes. Our detailed exploration into the microbial composition at various taxonomic levels, as illustrated in Supplementary Figure 1.

**FIGURE 3:**
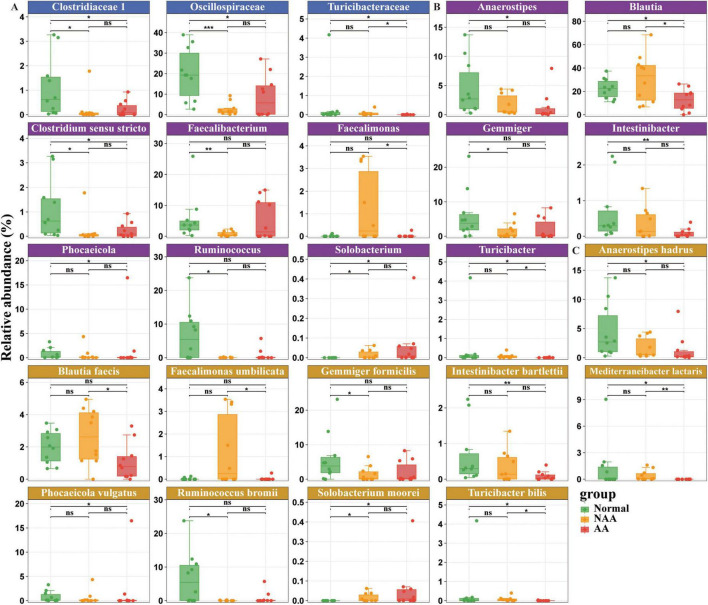
Differential gut microbiota composition at family, genus, species level between normal, NAA, and AA groups. **(A)** Family level. **(B)** Genus level. **(C)** Species level. Normal, normal group; NAA, non-advanced adenoma group; AA, advanced stage adenoma group. The data are presented as means ± SD, *n* = 10. *P < 0.05; **P < 0.01; ***P < 0.001.

At the family level (Figure 3A), the fecal microbiota primarily comprised three bacterial families: *Clostridiaceae 1, Oscillospiraceae*, and *Turicibacteraceae.* Compared to the NAA and AA groups, the gut microbiota of the normal group was enriched with *Clostridiaceae 1* and *Oscillospiraceae*. Furthermore, compared to the AA group, both the normal and NAA groups exhibited enrichment of *Turicibacteraceae* in the gut microbiome.

At the genus level (Figure 3B), the fecal microbiota primarily comprised bacteria from the following genera: *Anaerostipes, Blautia, Clostridium sensu stricto, Faecalibacterium, Faecalimonas, Gemmiger, Intestinibacter, Phocaeicola, Ruminococcus, Solobacterium*, and *Turicibacter*. Compared to the NAA group, the gut microbiota of the normal group was enriched with *Clostridium sensu stricto, Faecalimonas, Gemmiger*, and *Ruminococcus*. In contrast, the NAA group shows an enrichment of *Solobacterium* when compared to the normal group. Compared to the AA group, the normal group’s gut microbiota is enriched with *Anaerostipes, Blautia, Clostridium sensu stricto, Intestinibacter, Phocaeicola*, and *Turicibacter*. Conversely, compared to the normal group, the AA group exhibits an enrichment of *Solobacterium*. Furthermore, compared to the AA group, the NAA group’s gut microbiota is enriched with *Blautia, Faecalimonas*, and *Turicibacter*.

At the species level (Figure 3C), the fecal microbiota primarily includes the following species: *Anaerostipes hadrus, Blautia faecis, Faecalimonas umbilicata, Gemmiger formicilis, Intestinibacter bartlettii, Mediterraneibacter lactaris, Phocaeicola vulgatus, Ruminococcus bromii, Solobacterium moorei*, and *Turicibacter bilis*. Compared to the NAA group, the gut microbiota of the normal group is enriched with *Gemmiger formicilis* and *Ruminococcus bromii*. Conversely, compared to the normal group, the NAA group shows an enrichment of *Solobacterium moorei*. Compared to the AA group, the normal group’s gut microbiota is enriched with *Anaerostipes hadrus, Intestinibacter bartlettii, Mediterraneibacter lactaris, Phocaeicola vulgatus*, and *Turicibacter bilis*. On the other hand, compared to the normal group, the AA group exhibited an enrichment of *Solobacterium moorei*. Furthermore, compared to the AA group, the NAA group’s gut microbiota is enriched with *Blautia faecis, Faecalimonas umbilicata, Mediterraneibacter lactaris*, and *Turicibacter bilis*.

### 3.3 Predicted KEGG functional pathways in the gut microbiome

**FIGURE 4:**
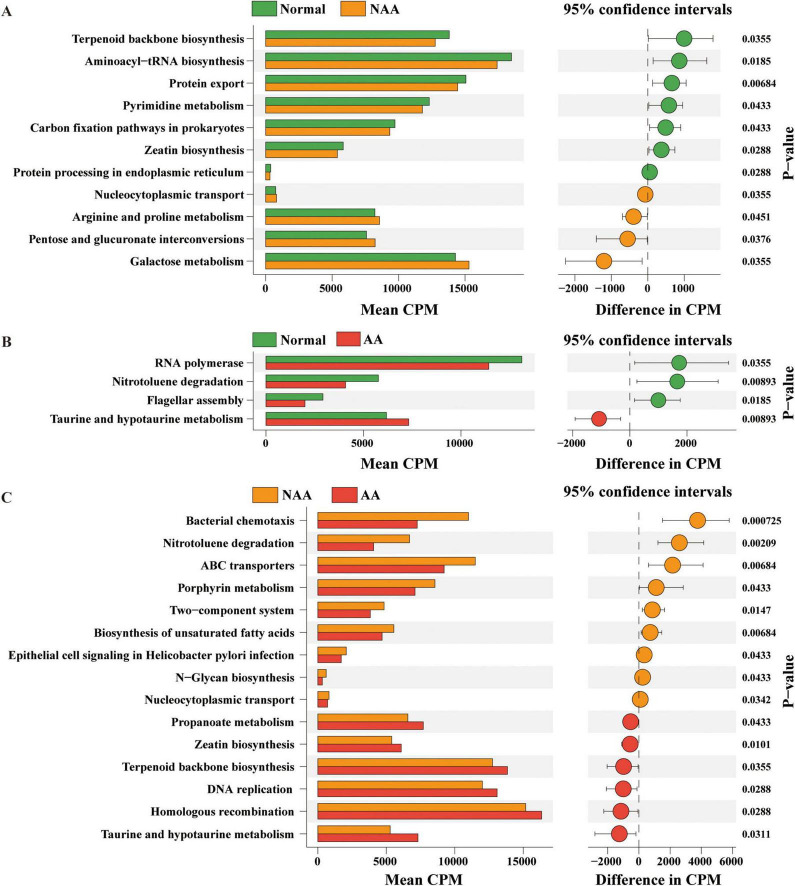
Kyoto Encyclopedia of Genes and Genomes (KEGG) functional pathways predicted in the gut microbiome of normal, NAA, and AA groups using Picrust2. **(A)** Linear discriminant analysis of predicted microbial metabolic functions between normal and NAA. **(B)** Linear discriminant analysis of predicted microbial metabolic functions between normal and AA. **(C)** Linear discriminant analysis of predicted microbial metabolic functions between NAA and AA. Selection of discriminative microbial pathways between groups were based on an LDA score cutoff of 3.0 and differences in the relative abundances of pathway (converted to log base 10) were statistically determined based on a Kruskal-Wallis and pairwise Wilcoxon tests. A *p*-value of <0.05 and a score ≥3.0 were considered significant in Kruskal-Wallis and pairwise Wilcoxon tests, respectively, at a significance level of 0.05; *n* = 10. Normal, normal group; NAA, non-advanced adenoma group; AA, advanced stage adenoma group.

In the normal vs. NAA comparison (Figure 4A), 11 KEGG pathways showed significant differences. The NAA group exhibited upregulation in *Nucleocytoplasmic transport, Arginine and proline metabolism, Pentose and glucuronate interconversions*, and *Galactose metabolism*. Downregulated pathways included *Terpenoid backbone biosynthesis, Aminoacyl-tRNA biosynthesis, Protein export, Pyrimidine metabolism, Carbon fixation pathways in prokaryotes, Zeatin biosynthesis, and Protein processing in the endoplasmic reticulum*.

In the normal vs. AA comparison (Figure 4B), three KEGG pathways showed significant differences. The AA group exhibited upregulation in *Taurine and hypotaurine metabolism* and downregulation in *RNA polymerase, Nitrotoluene degradation, and Flagellar assembly*.

In the NAA vs. AA comparison (Figure 4C), 15 KEGG pathways showed significant differences. The AA group exhibited upregulation in *Propanoate metabolism, Zeatin biosynthesis, Terpenoid backbone biosynthesis, DNA replication, Homologous recombination, and Taurine and hypotaurine metabolism*. Downregulated pathways included *Bacterial chemotaxis, Nitrotoluene degradation, ABC transporters, Porphyrin metabolism, Two-component system, Biosynthesis of unsaturated fatty acids, Epithelial cell signaling in Helicobacter pylori infection, N-Glycan biosynthesis, and Nucleocytoplasmic transport*.

### 3.4 Correlations between the microbial pathways and gut microbes

**FIGURE 5:**
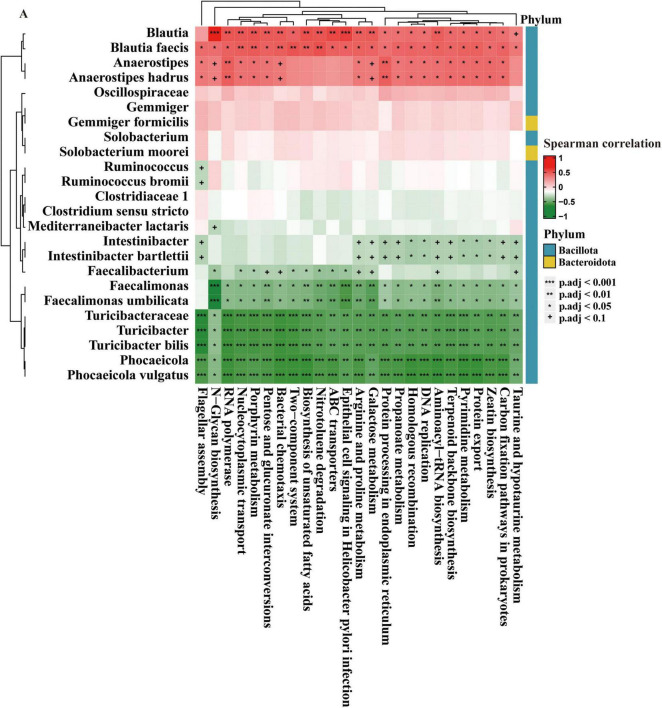
Correlation of gut microbes and microbial pathways. The data are presented as means ± SD, *n* = 10. +*P* < 0.1; *P < 0.05; **P < 0.01; ***P < 0.001.

Figure 5 provides an analysis of the correlations between various bacterial taxa and functional metabolic pathways in the gut microbiota at different stages of CRA (normal, NAA, AA). The heatmap organizes these taxa into two phyla, *Bacillota* and *Bacteroidota*. Significant positive correlations are observed for taxa like *Blautia, Blautia faecis, Anaerostipes*, and *Anaerostipes hadrus* with metabolic pathways such as Taurine and hypotaurine metabolism, Propanoate metabolism, and Zeatin biosynthesis. On the other hand, taxa such as *Faecalimonas, Faecalimonas umbilicata, Turicibacteraceae, Turicibacter*, and *Turicibacter bilis* are negatively correlated with pathways including Aminoacyl-tRNA biosynthesis, Terpenoid backbone biosynthesis, and Flagellar assembly.

### 3.5 PD-L1 expression and correlation of IL-6, IFN-γ, and gut microbes

**FIGURE 6:**
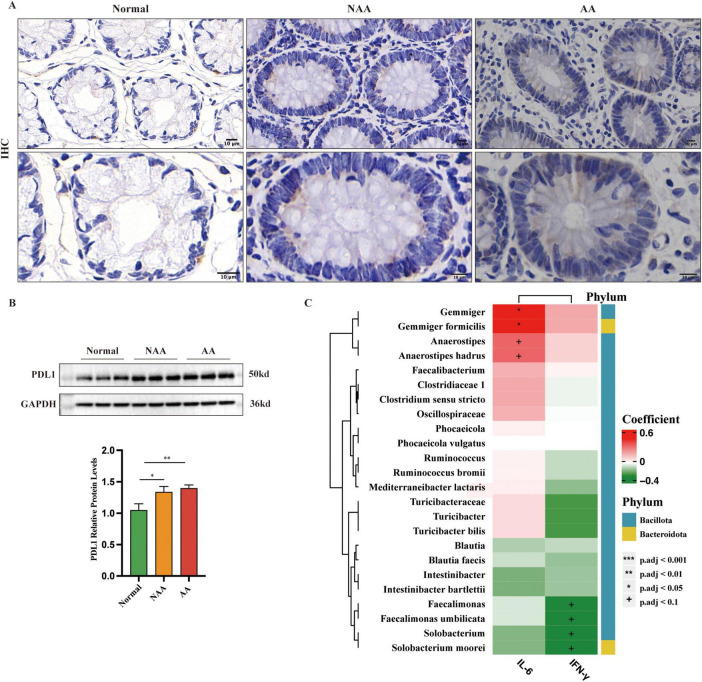
Expression of PDL1 as detected by immunohistochemistry (IHC) and western blot and correlations between the abundance of IL-6, IFN-γ and gut microbes. **(A)**. IHC stained sections from each of the normal, NAA, and AA groups. Membranous or cytoplasmic staining in shades of brown or yellow-brown indicates positive PD-L1 expression (100×, 200× magnification; scale bar: 10 μm). **(B)** Protein expression of PDL1 as detected by western blot (*n* = 5). **(C)** Correlations between the abundance of IL-6, IFN-γ and gut microbes. Normal, normal group; NAA, non-advanced adenoma group; AA, advanced stage adenoma group. Data were analyzed using one-way ANOVA. The data are presented as means ± SD, *n* = 10. +*P* < 0.1; *P < 0.05; **P < 0.01; ***P < 0.001.

Immunohistochemistry revealed increasing PD-L1 expression across the groups: normal (+), NAA (++), and AA (+++) (Figure 6A). Western blot analysis confirmed this trend, showing a progressive rise in PD-L1 levels from normal to AA (Figure 6B). Negative correlations were found between IFN-γ and *Faecalimonas, Faecalimonas umbilicata, Solobacterium*, and *Solobacterium moorei* (*p* < 0.1). Positive correlations were identified between IL-6 and *Gemmiger, Gemmiger formicilis, Anaerostipes*, and *Anaerostipes hadrus* (*p* < 0.05 for *Gemmiger* and *Gemmiger formicilis*, *p* < 0.1 for others) (Figure 6C).

## 4 Discussion

This study adds to the growing body of evidence linking the gut microbiome to CRC, with a specific focus on the precancerous stage of adenomas. By examining tissue PD-L1 protein expression, serum levels of inflammatory cytokines (IL-6, IFN-γ), and fecal gut microbiota composition across various stages of CRA, including normal, NAA, and AA, our research aimed to identify biomarkers indicative of significant changes during adenoma progression. Our research aims to identify biomarkers that signify important changes during adenoma progression and provide scientific support for dietary interventions by elucidating the functional roles of microbial taxa involved.

The observed reduction in α-diversity within the fecal microbiome associated with adenomas aligns with findings from Peters et al. (2016) who reported similar trends. However, contrasting results from Feng et al. (2015) study, which showed higher diversity in pre-tumor and tumor groups, highlight the complex nature of microbiome alterations during adenoma development. This discrepancy suggests that the relationship between microbiota diversity and adenoma progression may be influenced by additional factors, such as genetic predispositions, environmental influences, and lifestyle choices, that warrant further investigation. Specifically, dietary habits are known to play a crucial role in shaping gut microbiota composition. For instance, a high-fiber diet may enhance the diversity of beneficial microbial populations (Yang et al., 2021), while a Western-style diet may lead to an overrepresentation of pro-inflammatory bacteria (Shin et al., 2021). These dietary factors likely contribute to the observed differences in microbiota diversity and may be a key factor in adenoma progression. Notably, participants with the highest dietary fiber intake, particularly from cereals and fruits, had a lower risk of colorectal adenoma and distal colon cancer (Kunzmann et al., 2015), underscoring its protective role in CRC prevention.

The gut microbiota in the normal group was characterized by the enrichment of *Anaerostipes*, *Blautia*, *Gemmiger*, and *Ruminococcus*. Among them, higher levels of *Gemmiger* are associated with Western dietary patterns, while its reduced abundance in CRC patients exhibiting symptoms like bloody stools suggests a potential influence of diet on its prevalence (Chénard et al., 2020; Hur et al., 2022). Similarly, the positive correlation between dietary fiber intake and the higher population of *Ruminococcus* highlights its role in the fermentation of complex carbohydrates, emphasizing the significant impact of diet on gut microbiota composition and colorectal health. Recent studies have further demonstrated that specific dietary fibers may particularly promote the growth of beneficial bacteria such as *Ruminococcus*, further reinforcing the critical role of diet in CRC prevention (Abell et al., 2008; Abell et al., 2011).

*Anaerostipes and Blautia* are recognized as key producers of short-chain fatty acids (SCFAs), including acetate and butyrate. Butyrate, as a primary energy source, plays a crucial role in promoting the proliferation and repair of intestinal epithelial cells and exhibits strong anti-inflammatory effects by suppressing the nuclear factor-κB (NF-κB) signaling pathway. These bacteria generate SCFAs through the metabolism of lactate and acetate, as well as the fermentation of sugars and dietary fibers, which are essential for maintaining intestinal barrier integrity and controlling inflammation (Katsaounou et al., 2023; Maki et al., 2022; Song et al., 2023). In addition, *Anaerostipes and Blautia* are positively correlated with several important metabolic pathways, such as *taurine and hypotaurine metabolism, propanoate metabolism, and zeatin biosynthesis*. Emerging evidence highlights the role of taurine metabolism in regulating bile acid composition and enhancing gut microbiota diversity, which are pivotal in preventing the progression of adenomas (Duszka, 2022; Xu et al., 2021).

The abundance of these bacteria in the normal group decreases significantly during the NAA stage, with *Blautia* initially showing a slight higher before a marked reduction in the AA stage. This pattern indicates a gradual loss of protective bacterial functions and metabolic activities as adenomas progress. Furthermore, recent studies suggest that the metabolic capacity of the gut microbiota may undergo compensatory enhancement during the early stages of adenoma but collapse in later stages due to the intensification of the inflammatory environment and metabolic dysregulation. These findings highlight the importance of early intervention, particularly through dietary modulation, to support the growth of *Anaerostipes and Blautia*. Such interventions may serve as potential strategies for preventing adenoma progression. These findings emphasize the importance of early intervention, particularly through dietary modulation, to promote the growth of *Anaerostipes* and *Blautia*. Such interventions could be effective strategies for preventing adenoma progression.

In contrast, the NAA and AA groups were characterized by an enrichment of *Solobacterium*. This bacterium was more prevalent in the NAA group compared to the normal group. Its elevation in adenomatous polyps and negative correlation with IFN-γ suggest that *Solobacterium* may promote polyp progression via the NF-κB signaling pathway, potentially contributing to chronic inflammation and compromised intestinal barriers (Yu et al., 2024). These findings align with recent studies indicating that *Solobacterium* induces pro-inflammatory responses, making it a promising target for microbiome-based therapeutic interventions aimed at preventing adenoma progression.

PD-L1, a critical factor in tumor immune escape by inhibiting T-cell function, was assessed across different stages of CRA development. While some studies report elevated PD-L1 in high-grade adenomas and early invasive cancers, others find lower levels in progressive adenomas compared to benign adenomas (Gatenbee et al., 2022; Miller et al., 2018). Our study confirmed that PD-L1 levels higher with adenoma progression, yet the expression dynamics from normal tissue to CRC remain unclear, necessitating further investigation. Additionally, we found negative associations between *Faecalimonas, Faecalimonas umbilicata, Solobacterium*, and *Solobacterium moorei* with IFN-γ, and positive associations of *Gemmiger, Gemmiger formicilis, Anaerostipes*, and *Anaerostipes hadrus* with IL-6. These mixed findings regarding PD-L1 suggest a complex regulatory environment within the tumor microenvironment that warrants further study. Understanding these dynamics could lead to more targeted immunotherapies for CRC, potentially improving patient outcomes.

The modulation of diet to encourage the growth of beneficial bacteria, such as *Anaerostipes* and *Blautia*, is a promising strategy for slowing the progression of CRA and enhancing colorectal health. However, the primary challenge in dietary therapy is determining how to implement effective dietary adjustments, particularly in the early stages. A crucial aspect of this is the development of personalized dietary interventions as part of individualized treatment. A core question in clinical practice is how to use diet to alter the composition of the gut microbiome. Certain dietary components—such as dietary fiber, antioxidants, and fatty acids—may affect different populations in varying ways. For example, dietary fiber promotes the growth of beneficial bacteria and strengthens intestinal barrier function, while antioxidant-rich foods can help reduce inflammation. Despite the evident potential of dietary interventions in preventing CRA and CRC, the clinical application of such therapies faces several challenges. In traditional healthcare systems, dietary therapy is often underappreciated, and there is a lack of comprehensive understanding among clinicians. Additionally, patient compliance presents a significant barrier to the widespread adoption of dietary interventions. Future research should focus on further clarifying the relationships between dietary components, gut microbiota, and metabolic pathways. It should also assess the impact of different dietary strategies on gut health and explore their practical applications in clinical treatment.

This study has several limitations that warrant consideration. First, the relatively small sample size and single-center design may introduce selection bias and restrict the generalizability of the findings. Second, the reliance on 16S rRNA sequencing, particularly the V3-V4 region, constrains the taxonomic resolution to the genus level for most taxa, with species-level identifications being inferred probabilistically rather than determined definitively. The KEGG functional pathway analysis in this study was conducted using PICRUSt2 predictions, which are computational inferences rather than direct measurements. To address these limitations, future studies should include larger, more diverse cohorts and utilize advanced sequencing technologies to improve taxonomic resolution. Additionally, further research should focus on elucidating the mechanistic pathways that link specific microbial taxa to adenoma progression, providing deeper insights into their functional roles.

## 5 Conclusion

The study emphasizes the critical role of the gut microbiota in colorectal adenoma progression, highlighting key microbial taxa and metabolic pathways as potential biomarkers for early detection and therapeutic intervention. Early dietary modulation to promote beneficial bacteria such as *Anaerostipes and Blautia* could serve as a promising strategy to mitigate adenoma progression and improve colorectal health.

## Data Availability

The original contributions presented in this study are included in this article/Supplementary material, further inquiries can be directed to the corresponding authors. Sequences analyzed in this study are available in GenBank with the accession number PRJNA1107827.
