# Supplementary material for: Influence of gut microbiota and immune markers in different stages of colorectal adenomas
Source: Front Microbiol. 2025 Apr 16;16:1556056. doi: 10.3389/fmicb.2025.1556056 (PMC12040870; doi:10.3389/fmicb.2025.1556056)
Supplement: Supplementary file 1 [file Data_Sheet_1.DOCX]

Supplementary Material

## Supplementary Table1

| **Group** | **location** |
| --- | --- |
| Normal1 | rectum |
| Normal2 | sigmoid colon |
| Normal3 | sigmoid colon |
| Normal4 | sigmoid colon |
| Normal5 | sigmoid colon |
| Normal6 | rectum |
| Normal7 | rectum |
| Normal8 | rectum |
| Normal9 | rectum |
| Normal10 | sigmoid colon |
| NAA1 | descending colon |
| NAA2 | sigmoid colon |
| NAA3 | ascending colon |
| NAA4 | sigmoid colon |
| NAA5 | ascending colon |
| NAA6 | sigmoid colon |
| NAA7 | sigmoid colon |
| NAA8 | sigmoid colon |
| NAA9 | sigmoid colon |
| NAA10 | transverse colon |
| AA1 | rectum |
| AA2 | rectum |
| AA3 | sigmoid colon |
| AA4 | transverse colon |
| AA5 | rectum |
| AA6 | sigmoid colon |
| AA7 | rectum |
| AA8 | rectum |
| AA9 | sigmoid colon |
| AA10 | sigmoid colon |

**Supplementary Table 1.** Normal: Normal group; NAA: Non-advanced adenoma group; AA: Advanced stage adenoma group.

## Supplementary Figures


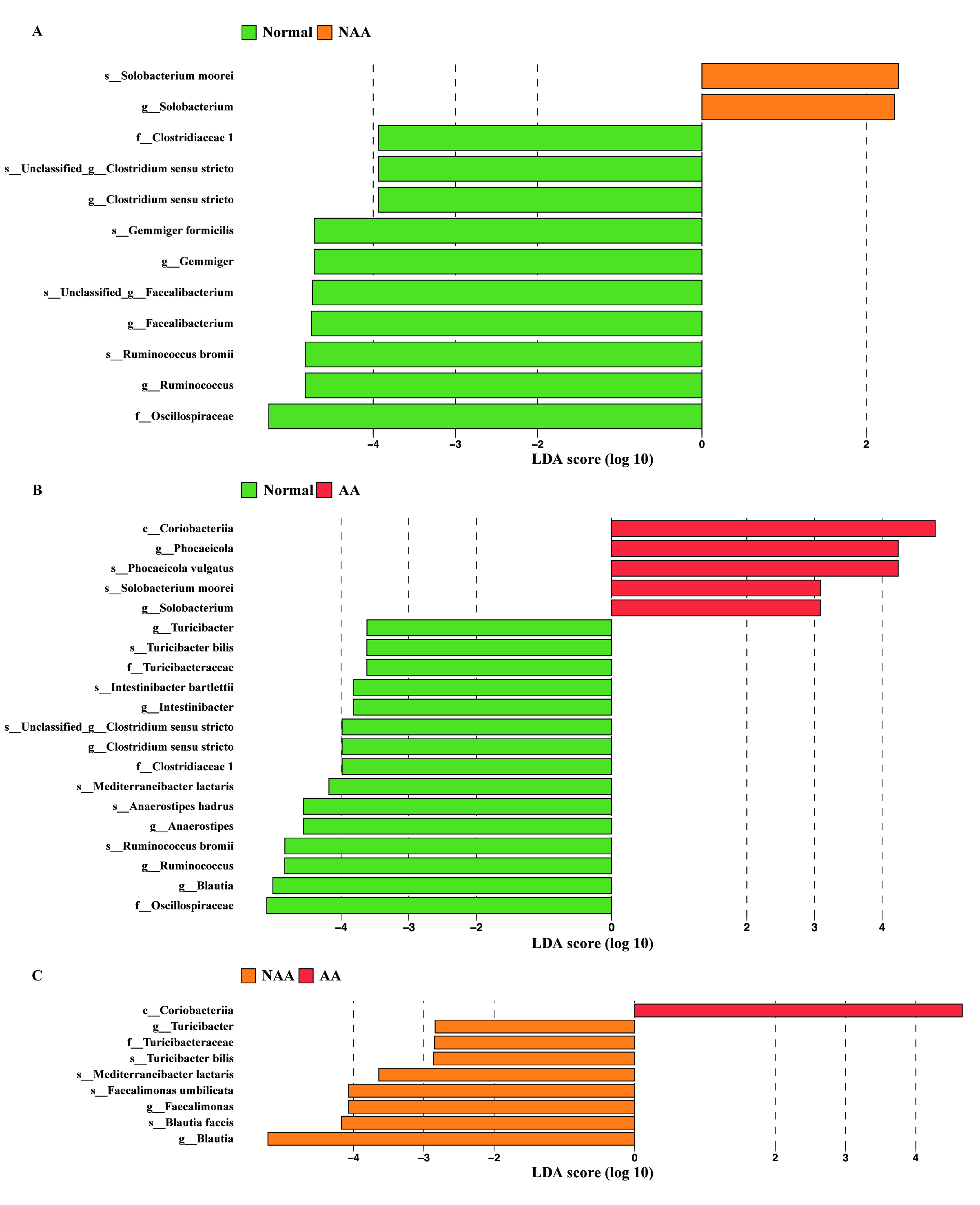


**Supplementary Figure 1.** Linear discriminant analysis. (A) Differential abundance of taxa was ranked according to their effect size between Normal and NAA. (B) Differential abundance of taxa was ranked according to their effect size between Normal and AA. (C) Differential abundance of taxa was ranked according to their effect size between NAA and AA. Selection of discriminative taxa between groups were based on an LDA score cutoff of 3.0 and differences in the relative abundances of taxa (converted to log base 10) were statistically determined based on a Kruskal–Wallis and pairwise Wilcoxon tests. A p-value of < 0.05 and a score ≥ 3.0 were considered significant in Kruskal-Wallis and pairwise Wilcoxon tests, respectively, at a significance level of 0.05; n = 10. The length of the histogram represents the LDA score; the degree of influence of species with significant difference between different groups.
