# Supplementary figures and images for: Influence of gut microbiota and immune markers in different stages of colorectal adenomas
Source: Front Microbiol. 2025 Apr 16;16:1556056. doi: 10.3389/fmicb.2025.1556056 (PMC12040870; doi:10.3389/fmicb.2025.1556056)

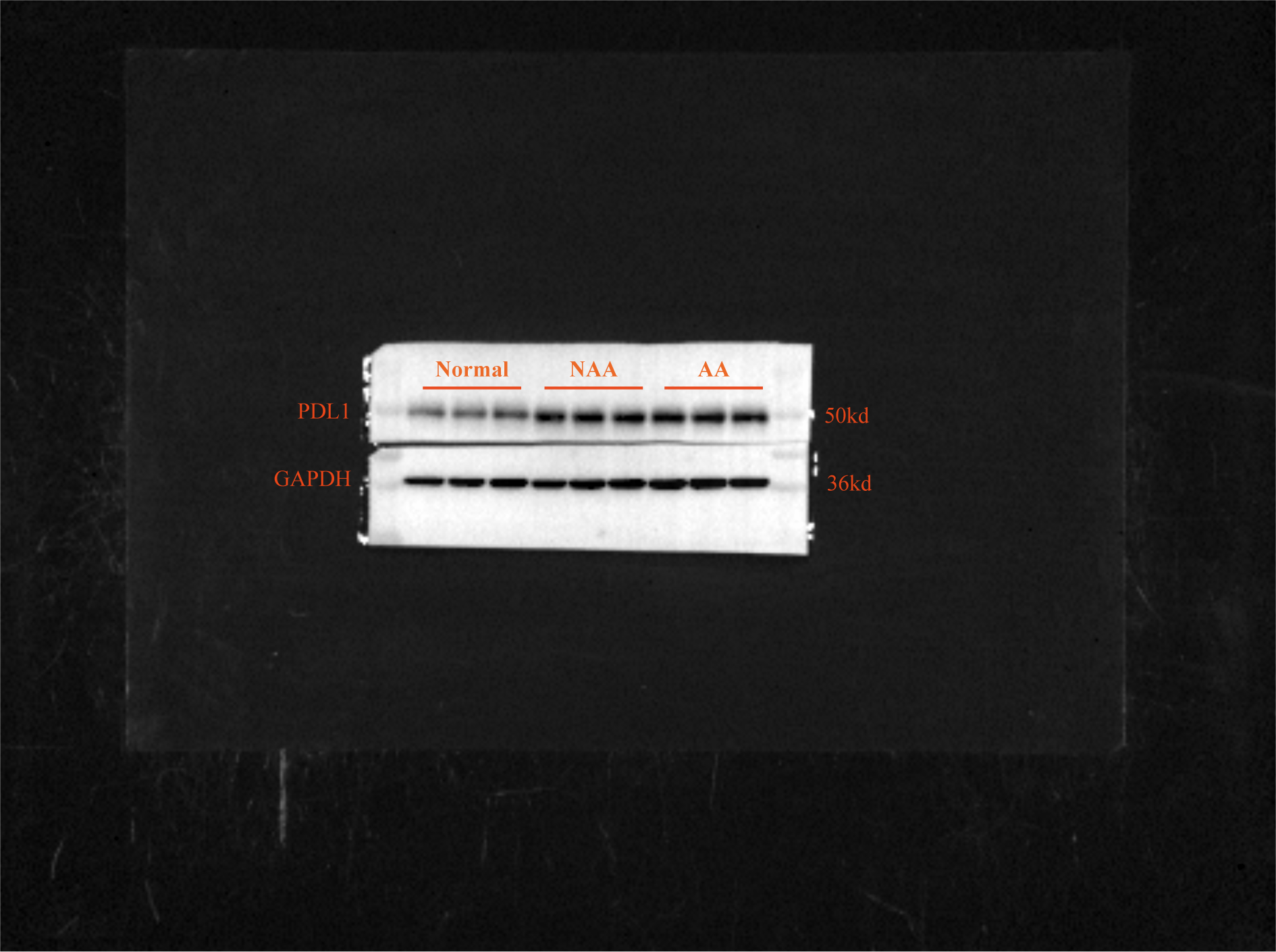

Supplement: Supplementary file 2 [file Image_1.TIF]
